# Examination of the Transcriptional Response to *LaMIR166a* Overexpression in *Larix kaempferi* (Lamb.) Carr

**DOI:** 10.3390/biology10070576

**Published:** 2021-06-23

**Authors:** Yanru Fan, Wanfeng Li, Zhexin Li, Shaofei Dang, Suying Han, Lifeng Zhang, Liwang Qi

**Affiliations:** 1 State Key Laboratory of Tree Genetics and Breeding, Research Institute of Forestry, Chinese Academy of Forestry, Beijing 100091, China; fanyanru@caf.ac.cn (Y.F.); liwf@caf.ac.cn (W.L.); forestry2016dang@163.com (S.D.); 2 Key Laboratory of Tree Breeding and Cultivation, National Forestry and Grassland Administration, Research Institute of Forestry, Chinese Academy of Forestry, Beijing 100091, China; 3 Chongqing Key Laboratory of Economic Plant Biotechnology, College of Landscape Architecture and Life Science/Institute of Special Plants, Chongqing University of Arts and Sciences, Chongqing 402160, China; lzx8903@cqwu.edu.cn; 4 Research Institute of Forest Ecology, Environment and Protection, Chinese Academy of Forestry, Beijing 100091, China; syhan@caf.ac.cn

**Keywords:** miR166a, HD-ZIP III, *Larix kaempferi* (Lamb.) Carr, transcriptome, correlation analysis

## Abstract

**Simple Summary:**

The study of somatic embryogenesis can provide insights into early plant development. To elucidate the molecular mechanisms associated with *miR166* in *Larix kaempferi* (Lamb.) Carr, the transcriptional profiles of wild-type (WT) and *LaMIR166a*-overexpressing embryonic cells were subjected to RNA sequencing. In total, 2467 differentially expressed genes were obtained. The cleaved degree of *LaHDZ31–34* was higher in transgenic lines than in WT. The genes related to *LaHDZ31–34* were screened by transcriptome analysis, and a yeast one-hybrid and dual-luciferase report assay revealed that LaHDZ31–34 could bind to the promoters of *LaPAP, LaPP1, LaZFP5,* and *LaPHO1.* This study provides insights into the regulatory mechanisms of *miR166*.

**Abstract:**

The study of somatic embryogenesis can provide insight into early plant development. We previously obtained *LaMIR166a*-overexpressing embryonic cell lines of *Larix kaempferi* (Lamb.) Carr. To further elucidate the molecular mechanisms associated with *miR166* in this species, the transcriptional profiles of wild-type (WT) and three *LaMIR166a*-overexpressing transgenic cell lines were subjected to RNA sequencing using the Illumina NovaSeq 6000 system. In total, 203,256 unigenes were generated using Trinity de novo assembly, and 2467 differentially expressed genes were obtained by comparing transgenic and WT lines. In addition, we analyzed the cleaved degree of *LaMIR166a* target genes *LaHDZ31–34* in different transgenic cell lines by detecting the expression pattern of *LaHdZ31–34*, and their cleaved degree in transgenic cell lines was higher than that in WT. The downstream genes of *LaHDZ31–34* were identified using Pearson correlation coefficients. Yeast one-hybrid and dual-luciferase report assays revealed that the transcription factors LaHDZ31–34 could bind to the promoters of *LaPAP*, *LaPP1*, *LaZFP5*, and *LaPHO1*. This is the first report of gene expression changes caused by *LaMIR166a* overexpression in Japanese larch. These findings lay a foundation for future studies on the regulatory mechanism of miR166.

## 1. Introduction

Japanese larch (*Larix kaempferi* (Lamb.) Carr) is an economically and ecologically important coniferous timber tree species in northern China [1]. Conventional breeding and genetic improvement techniques can no longer satisfy the national cultivation demand for this tree. Propagation by somatic embryogenesis, which is based on cellular totipotency, is conducive to the rapid breeding and large-scale reproduction of high-quality Japanese larch strains. Furthermore, somatic embryogenesis is an ideal technique for studying the growth and development of gymnosperms.

MicroRNAs (miRNAs) are 19–24-nucleotide-long single-stranded RNAs that regulate target mRNAs, affecting their translation [2,3]. *miR165* and *miR166* are among the most abundant and highly conserved miRNAs in terrestrial plants, performing regulatory functions in plant development by specifically clipping and inhibiting the expression of class III homeodomain leucine zipper (HD-ZIP III) [4,5,6]. The *Arabidopsis* genome encodes seven copies of *miR166* and two of *miR165*, which produce conserved sequences with the transcript of the *HD-ZIP III* genes [7]. The *miR165/166* members and *HD-ZIP III* genes are known to regulate plant growth and development processes. In *Arabidopsis thaliana*, the HD-ZIP III family includes five genes, *ATHB8*, *ATHB15*, *phavoluta* (*PHV*), *phabulosa* (*PHB*), and revoluta (*REV*), which determine and direct the differentiation and maintenance of stem apex meristem cells, the apical meristem, vascular bundle development, and organ polarity [8,9]. An analysis of the expression of the *HD-ZIP III* genes *PpHB14*, *PpHB15*, *PpHB8*, and *PpREV* in peach (*Prunus persica*) tissue revealed that they are regulated by *miR166* during fruit development [10]. As plant-specific transcription factors (TFs), the HD-ZIP III TFs regulate plant development and auxin-related gene expression [11,12]. Furthermore, they play key roles in the development from embryo to maturity in *Arabidopsis* and other plants [13,14]. HD-ZIP III regulates vascular patterning, meristem structure, and adaxial identity in *Nicotiana sylvestris* [15]. The overexpression of various *miR166* genes has different effects on the expression of the *HD-ZIP III* genes; in *Arabidopsis*, *PHB*, *PHV*, and *ATHB15* are downregulated, whereas *REV*, Meristem enlarged1 (*men1*), and jabba-1D *(jab-1D)* are upregulated. This difference in the effects can be attributed to a dose-dependent interaction between *miR166* and *HD-ZIP III* [16,17]. *PHB* expression is affected by the zinc finger protein SERRATE, which affects leaf axis patterning in *Arabidopsis* [18].

Most studies on the effects of *miR166a* on plant growth regulation and development have been in *Arabidopsis*, rice, and other angiosperms, but rarely on gymnosperms. We previously created five *LaMIR166a*-overexpressing cell lines to study the function of *miR166* [19]. Here, we analyzed the transcriptome of *LaMIR166a*-overexpressing embryogenic suspensor masses (ESMs) and the expression pattern of *LaHDZ31–34* in wild-type (WT) and transgenic lines by quantitative real-time reverse-transcription PCR (qRT-PCR). We identified several genes that were affected by *LaMIR166a* overexpression by transcriptome sequencing and analyzed their promoters. The promoters containing the binding sites of LaHDZ31–34 were cloned to construct a yeast one-hybrid (Y1H) library, revealing that LaHDZ31–34 can bind to gene promoters. The Y1H and dual-luciferase (LUC) report assay results can provide a basis for further research on the functions of HD-ZIP III. This study is expected to enrich the transcriptome information available for *L. kaempferi*; examine the roles, regulatory mechanisms, and expression of *miR166a* and its target gene (*HD-ZIP III*) in ESMs; and provide valuable insights into the regulatory network function of *miR166*–*HD-ZIP III*.

## 2. Materials and Methods

### 2.1. Plant Materials

Embryogenic cultures were induced from *L. kaempferi* immature zygotic embryos. Previously, *LaMIR166a* has been cloned into the pSuper1300 (+) binary vector and successfully transformed into WT ESMs, resulting in transgenic cell lines named a-1 to a-5 [19]. Here, three biological replicates were obtained from a-3, a-4, and a-5. Four embryogenic cultures without transformation were used as the control. The materials obtained from media were immediately frozen in liquid nitrogen and stored at −80 °C until RNA extraction.

### 2.2. RNA Preparation and Detection

The total RNA was isolated using the RNAiso Plus and RNAiso-mate for Plant Tissue kits (Takara, Japan), according to the manufacturer’s instructions. The total RNA was treated with DNase (Takara, Japan) to remove DNA. To ensure the accuracy of the sequencing data, the total RNA samples were prepared as follows. First, RNA purity was detected based on the OD 260/280 ratio using a NanoDrop 1000 Spectrophotometer (Thermo Scientific, Waltham, MA, USA). Thereafter, agarose gel electrophoresis was performed to analyze the extent of RNA degradation and to detect contamination. Furthermore, the RNA concentration was precisely quantified using a Qubit fluorometer. Finally, the Agilent 2100 system was used to detect RNA integrity.

### 2.3. Library Preparation, RNA Sequencing, and Data Quality Control

Thirteen RNA libraries were constructed and sequenced on an Illumina NovaSeq 6000 system (Berry Genomics, China). Raw reads, which affect the alignment and quality of the subsequent analyses, were filtered to obtain clean reads. Spliced, repetitive, and low-quality reads (mass value Q ≤ 3) and those with unknown bases accounting for >50% of the total reads were removed to obtain more reliable results. 

### 2.4. De Novo Assembly and Gene Functional Annotation

De novo assembly of *L. kaempferi* transcriptome was performed using Trinity v. 2.4.0 [20]. The number and length of unigenes and their GC content were determined after assembly. Gene function was annotated by a BLAST (e < 10^−5^) search against the following databases: NCBI nucleotide sequences (Nt, ftp://ftp.ncbi.nlm.nih.gov/blast/db/, accessed on 21 April 2021), NCBI non-redundant protein sequences (Nr, ftp://ftp.ncbi.nlm.nih.gov/blast/db/, accessed on 21 April 2021), Swiss-Prot (a manually annotated and reviewed protein sequence database, http://www.ebi.ac.uk/uniprot, accessed on 21 April 2021), Kyoto Encyclopedia of Genes and Genomes (KEGG, http://www.genome.jp/kegg/, accessed on 21 April 2021), Gene Ontology (GO, http://geneontology.org/, accessed on 21 April 2021), and Eukaryotic Ortholog Groups (KOG, ftp://ftp.ncbi.nlm.nih.gov/pub/COG/, accessed on 21 April 2021).

### 2.5. Differentially Expressed Genes and Pathway Enrichment Analysis

The expression of unigenes is affected by samples or experimental conditions. EdgeR software was used to determine the *p*-value and false discovery rate (FDR) for the differentially expressed unigenes in each sample, based on the alignment results. FDR and fold-change (FC) values were used to screen differentially expressed transcripts; transcripts with an FC ≥ 1 and FDR < 0.05 in a comparison were considered significantly differentially expressed [21]. Differentially expressed genes (DEGs) were classified and grouped using the GO and KEGG pathway analyses to identify the associated biological pathways in the WT and *LaMIR166a*-overexpressing lines. Significance threshold was set at *p* < 0.05.

### 2.6. LaHDZ31–34 Expression Patterns Detected by Quantitative RT-PCR

One microgram of RNA was reverse transcribed into cDNAs using the TranScript All-in-One First-Strand cDNA Synthesis Supermix for qPCR (TransGen Biotech, Beijing, China). cDNAs were diluted to a suitable concentration, and 2 μL of the cDNA solution was used to detect *LaHDZ31–34* expression by qRT-PCR, using the TB Green Premix Ex Taq kit (Tli RNase H Plus; Takara, Japan). Reactions were performed on a 7300 Real-Time PCR System (Applied Biosystems, Forest System, CA, USA). All gene expression levels were normalized to those of an internal control, *LaEF1A1* (JX157845) [19]. The results were based on an average of three biological replicates, and they are shown as the mean ± SD.

The cleaved degree of target genes can be measured by investigating the relative expression levels of the initial transcript (both non-cleaved and cleaved transcripts) and of the full-length transcript (non-cleaved transcripts). The initial transcript was used as an internal control to calculate the cycle threshold (|Ct|) for the full-length transcript. The larger the |Ct| value, the greater the cleaved degree [22]. According to this principle, two primers were used to investigate the cleaved degree of *LaHDZ31–34.* Initial mRNA transcript primers were located downstream from the *LaMIR166a* cleavage sites, and non-cleaved mRNA transcript primers spanned the *LaMIR166a* target site in each gene. The primers used are shown in Table S7.

### 2.7. Discovery of the LaHDZ31–34 Candidate Genes in Response to LaMIR166a Overexpression in L. kaempferi

To identify candidate genes responding to LaHDZ31–34 protein deficiency in the *LaMIR166a*-overexpression lines, the expression profiles of *LaHDZ31–34* based on transcriptome data were analyzed in different cell lines. Using BioEdit v.7.1.11, we uploaded the local transcriptome sequence database for the 13 samples, with *LaHDZ31–34* coding sequences [19] as the subsequences, to determine the transcriptome sequence ID by local BLAST alignment.

Candidate genes were hierarchically clustered on the basis of Pearson’s correlation coefficients (r). The putative genes were those with expression patterns highly similar to those of *LaHDZ31–34* in response to *LaMIR166a* overexpression. Pearson’s correlation coefficients between the mean fragments per kilobase of transcript per million mapped reads of *LaHDZ31–34* and DEGs corresponding to each *LaMIR166a*-overexpression cell line were calculated. Significantly co-expressed genes were those with |r| > 0.9. The related genes of the four TFs LaHDZ31–34 were analyzed using a Venn diagram.

### 2.8. Cis-Elements in Promoter Prediction

Promoters for the related genes were obtained from *L. kaempferi* genome data (https://www.ncbi.nlm.nih.gov/genome/12799, accessed on 21 April 2021); the genome sequence number corresponding to the transcriptome is shown in Table S8. Potential *cis*-acting regulatory elements related to *LaHDZ31–34* in the sequences, approximately 3000 bp upstream of the translational start site (ATG), were investigated using the online database PlantCARE (http://bioinformatics.psb.ugent.be/webtools/plantcare/html/, accessed on 21 April 2021).

### 2.9. Cloning of Related Gene Promoters and Y1H, Dual-Luciferase Report Assay

Genomic DNA, obtained from the ESMs as templates, was purified using the Plant Genomic DNA Extraction Kit (BioTeke, Wuxi, China) following the manufacturer’s protocol. Promoter sequences of the related genes were obtained by PCR using genomic DNA. High-fidelity ExTaq DNA Polymerase (Takara, Japan) was used in PCR, and gene-specific primers were designed using Primer 5.0 (Premier Biosoft International, Palo Alto, CA, USA). The full-length open reading frames of the TFs *LaHDZ31–34* were cloned using cDNA as described in Section 2.6. The primers for promoters and TFs are shown in Table S9. All PCR products were cloned into the pBM23A vectors (BioMed, Shanghai, China) and transformed into *Escherichia coli* DH5a strain. At least five clones per gene were randomly selected and sequenced.

The promoters were inserted into the pHIS2 vector. The TF genes *LaHDZ31–34* were inserted into the pGADT7 AD vector; *LaHDZ31* was inserted into BamHI and EcoRI sites, and *LaHDZ33* was inserted into the ClaI and *Eco*RI restriction sites of pGADT7. *LaHDZ32* and *LaHDZ34* were inserted into the same SacI and EcoRI sites of pGADT7 to generate the effector vectors (Table S9). The Y1H assay was used to examine the downstream target gene of *LaHDZ31–34*. The plasmid pGADT7-LaHDZ31–34 and pHIS2-related gene promoters were transformed into yeast Y187 via lithium acetate transformation. The yeast cells were cultured for 3 d on SD/-Leu/-Trp medium and incubated in a 28 °C incubator. The three positive clones were then selected from the SD/-Leu/-Trp medium and transferred onto SD/-Leu/-Trp/-His medium containing 30 mmol/L 3-amino-1,2,4-triazole (3-AT). Each clone had two spots.

The relationship between *LaHDZ31–34* and related genes was further confirmed by dual-luciferase (LUC) assays. The promoter fragments were cloned into the pGreenII 0800-LUC vector as reporters and *LaHDZ31–34* were cloned into the pGreenII 0029 62-SK vector as effectors (Table S10). Then, effectors and reporters were transformed into *Agrobacterium tumefaciens* strain GV3101. The mixed *Agrobacterium* cells were infiltrated into 4-week-old tobacco (*N. benthamiana*) leaves. At 72 h after infiltration, the activity of promoters was determined by calculating the ratio of firefly luciferase (LUC) to *Renilla luciferase* (REN) using the Dual-Luciferase Reporter Assay System (Promega, Madison, WI, USA).

## 3. Results

### 3.1. De Novo Transcriptome Assembly and Gene Expression Profiles 

**Figure 1:**
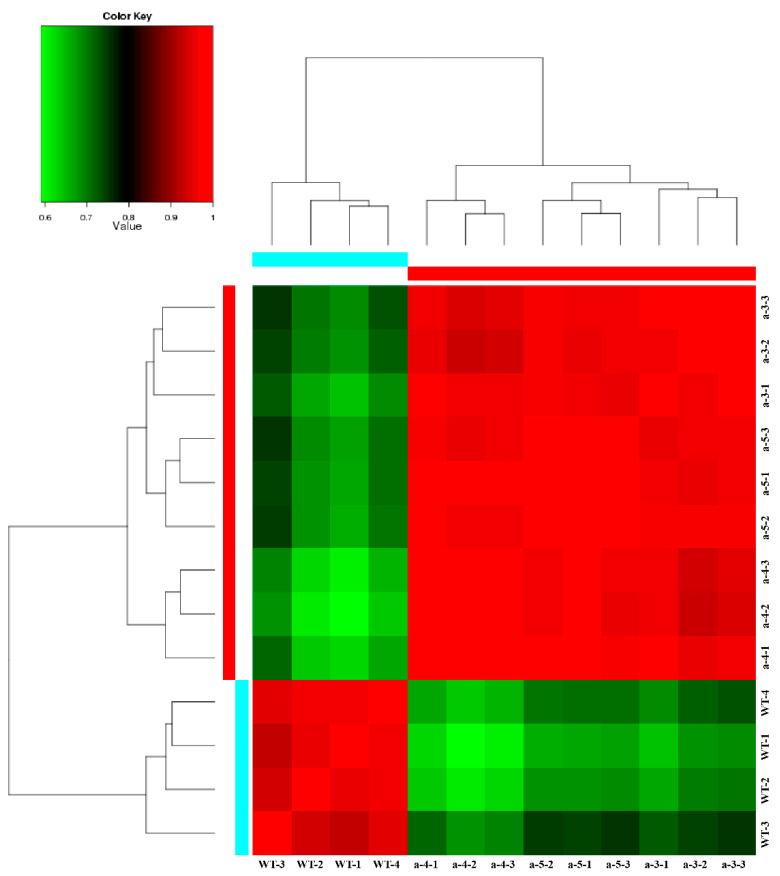
Pearson’s correlation coefficients among the samples. WT represents wild embryogenic cultures and a represents transgenic embryogenic cultures, including a-3, a-4, and a-5.

To better understand the function of *LaMIR166a,* we performed transcriptomic analysis using wild-type and MIR166a-overexpressing cell lines. The raw sequencing data have been submitted to the National Center for Biotechnology Information (NCBI) Sequence Read Archive (http://www.ncbi.nlm.nih.gov, accessed on 21 April 2021, accession number: PRJNA680899). A total of 6.30 Gb of clean data was obtained for each sample, and the quality of the clean reads is presented in Table 1. The clean data were assembled into 203,256 non-redundant unigenes, with N50 length of approximately 996 bp (Table 1); correlation could be observed between samples (Figure 1).

### 3.2. Gene Function Annotation

In total, 203,256 unigenes were annotated to one or more functions using the GO, KEGG, KOG, NR, NT, and Swiss-Prot databases. After eliminating redundancy from different databases, 58,487 unigenes were annotated at least once (Table 2).

**Figure 2:**
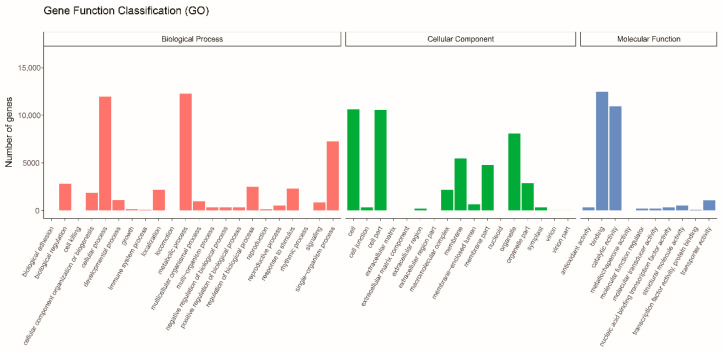
Gene ontology classification of *Larix kaempferi* transcriptome. Unigenes were annotated to three categories: Biological Process (red), Cellular Component (green), and Molecular Function (blue).

**Figure 3:**
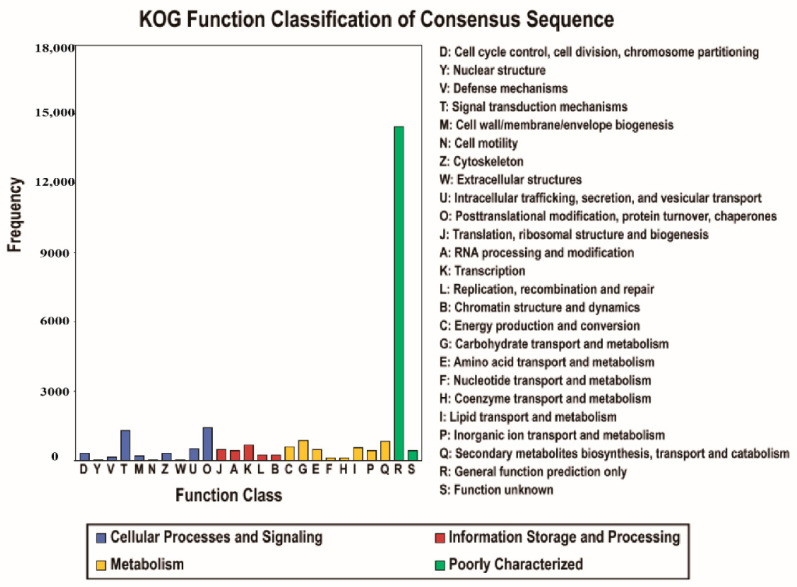
Eukaryotic Ortholog Groups (KOG) functional classification of consensus sequence for *Larix kaempferi.* In total, 23,331 unigenes were distributed in 25 KOG function classes.

**Figure 4:**
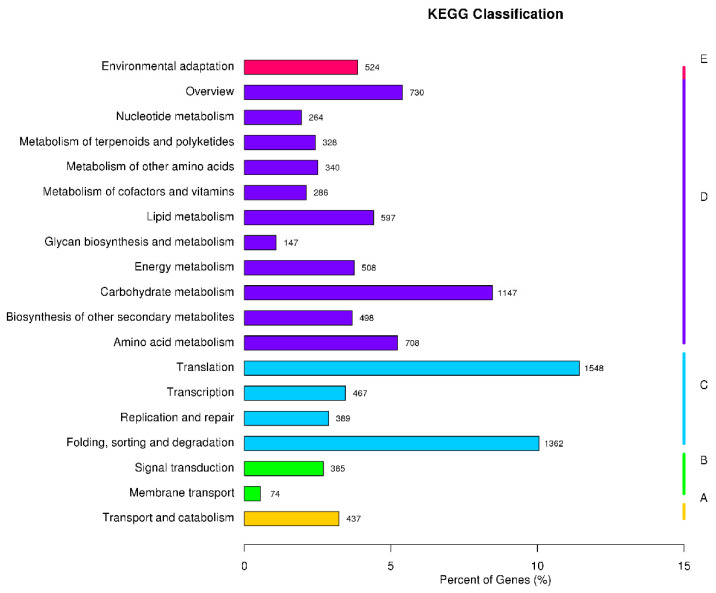
Kyoto Encyclopedia of Genes and Genomes (KEGG) classification of *Larix kaempferi* transcriptome. The numbers after the bars indicate the number of genes annotated to the pathway. The genes were divided into five branches according to the KEGG metabolic pathway: (**A**) cellular processes, (**B**) environmental information processing, (**C**) genetic information processing, (**D**) metabolism, and (**E**) organismal systems.

In the GO database, 26,234 unigenes were annotated at four levels (Figure 2). The unigenes were classified into 49 subcategories within three standard categories: biological process (BP), cellular component (CC), and molecular function (MF). The most enriched terms in the BP, CC, and MF domains were “metabolic process,” “cell,” and “binding”, respectively. In the KOG database, 23,331 unigenes were annotated into 25 groups (Figure 3). “General function prediction only” was the largest group, followed by “posttranslational modification, protein turnover, chaperones” and “signal transduction mechanisms”. In the KEGG database, 13,546 unigenes were assigned to 129 KEGG pathways (Table S1). The genes were divided into five branches according to the KEGG metabolic pathway involved as follows: (A) cellular processes, (B) environmental information processing, (C) genetic information processing, (D) metabolism, and (E) organismal systems (Figure 4). In the “biosynthesis of other secondary metabolites” category, 498 unigenes were annotated to 12 KEGG secondary metabolites; “phenylpropanoid biosynthesis” was the largest group, with 239 unigenes, followed by “flavonoid biosynthesis” with 133 unigenes, and “flavone and flavonol biosynthesis”.

### 3.3. Screening DEGs between WT and Transgenic Lines 

**Figure 5:**
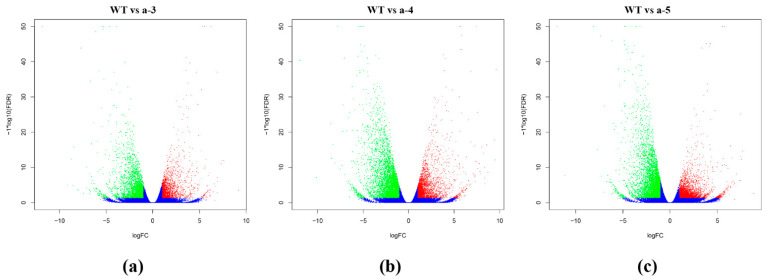
Volcano plots of differentially expressed genes (DEGs) among the wild-type (WT), a-3, a-4, and a-5 lines, based on transcriptomic analysis of L*arix kaempferi*. The plots compare gene expression between the WT and (**a**) a-3, (**b**) a-4, and (**c**) a-5. Upregulated DEGs in the upper right (red), downregulated DEGs in the upper left (green), and nondifferentially accumulated genes at the bottom (blue).

**Figure 6:**
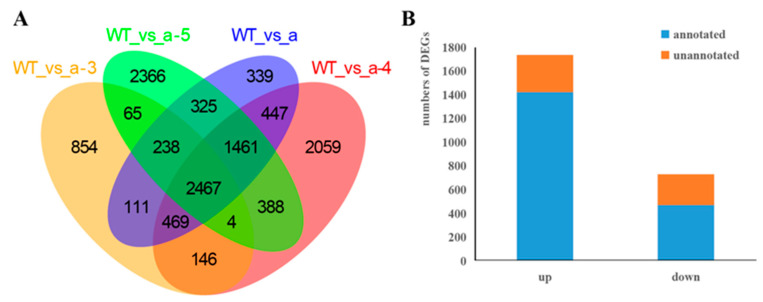
Statistical analysis of DEGs among the WT, a-3, a-4, and a-5 lines, based on the transcriptomic analysis of *Larix kaempferi*. (**A**) Venn diagram of DEGs common to the WT, a-3, a-4, and a-5. (**B**) The numbers of upregulated and downregulated DEGs.

The volcano plots show the differences among the WT and transgenic groups (Figure 5). The Venn diagram analysis revealed 2467 DEGs in the a-3, a-4, a-5, a, and WT groups (Figure 6A, Table S2). Contrasting the WT against the cell lines is equivalent to comparing the differences between the control and experimental group (calculated based on three technical duplicates: a-3, a-4, and a-5), which produces fewer DEGs and is thereby conducive to subsequent validation and functional studies. The largest difference in gene expression between the WT and transgenic lines was detected for the hygromycin resistance protein gene (|log_2_ (FC)| = 11.9), which was not expressed in the WT. The hygromycin resistance protein gene is related to the carriers used in the transformation of cell lines [19].

In total, 1424 genes were annotated from the 1736 upregulated DEGs and 473 genes were annotated from 731 downregulated DEGs (Figure 6B). The number of DEGs between each of the *LaMIR166a*-overexpressing transgenic lines and the WT differed (Table 3). This reveals that the transgenic strains vary in gene expression and that molecular regulation mechanisms should be studied according to the expression of target genes.

### 3.4. GO Enrichment and KEGG Pathway Analysis of DEGs

The GO and KEGG analyses were conducted to further identify the MFs and BPs associated with the DEGs. “Secondary metabolite biosynthetic process” (GO: 0044550) was the most significantly enriched term in the BP category, “zinc ion” (GO: 0008270) was the most significantly enriched in the MF category, and “membrane” (GO: 0016020) was highly enriched in the CC category (Table S3). These findings suggest that *LaMIR166a* overexpression might influence the expression of genes related to metabolite biosynthesis in ESMs. 

**Figure 7:**
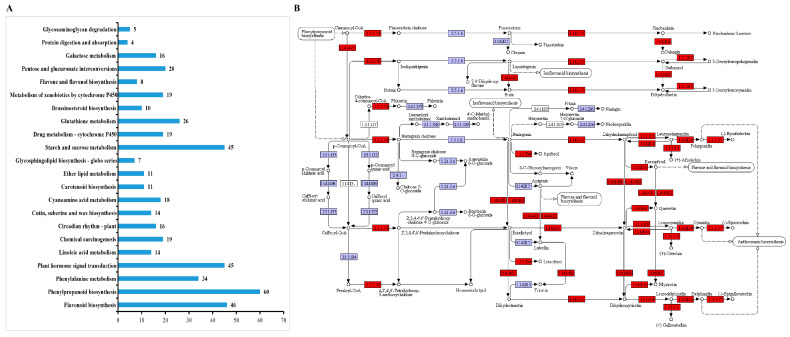
Kyoto Encyclopedia of Genes and Genomes (KEGG) pathway analysis of *Larix kaempferi* transcriptome sequencing data. (**A**) Overview of the KEGG analysis results (*p* < 0.05). (**B**) Genes with considerable marked changes (*p* < 0.05) in the expression of flavonoid biosynthesis pathways, in the *LaMIR166a*-overexpressing lines.

In total, 1088 DEGs were mapped to 229 KEGG terms (Table S4). The KEGG enrichment analysis of these data revealed 22 enriched metabolic pathways (*p* < 0.05). Of these, two pathways closely related to flavonoid biosynthesis and accumulation, namely ko00940 (phenylpropanoid biosynthesis) and ko00941 (flavonoid biosynthesis), were significantly enriched (Figure 7A). The BPs related to flavonoid accumulation were identified; the changes in gene expression in the colored group are shown in Figure 7B. Genes involved in flavonoid biosynthesis, such as phenylalanine ammonia-lyase (*PAL*), chalcone synthase (*CHS*), dihydroflavonol-4-reductase (*DFR*), leucoanthocyanidin dioxygenase (*ANS*), flavonol synthase (*FLS*), and flavanone 3-hydroxylase (*F3H*), were upregulated relative to their levels in the WT.

### 3.5. LaHDZ31–34 Expression Patterns 

**Figure 8:**
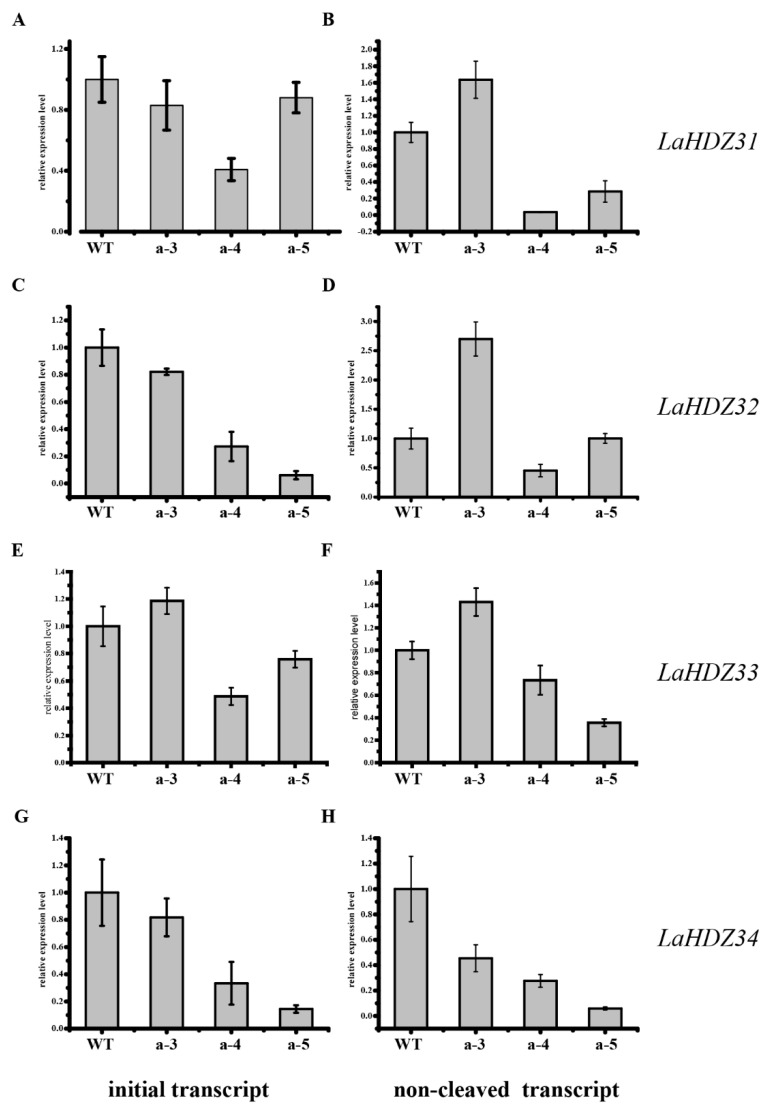
Analysis of the cleaved degree of *LaHDZ31–34*. (**A**) Expression patterns of initial transcripts of *LaHDZ31* in the wild-type (WT), a-3, a-4, and a-5 lines. (**B**) Expression patterns of the non-cleaved transcripts of *LaHDZ31* in the WT, a-3, a-4, and a-5. Expression patterns of the (**C**) initial transcripts of *LaHDZ32* in WT, a-3, a-4, and a-5; (**D**) non-cleaved transcripts of *LaHDZ32* in WT, a-3, a-4, and a-5; (**E**) initial transcripts of *LaHDZ33* in WT, a-3, a-4, and a-5; (**F**) non-cleaved transcripts of *LaHDZ33* in WT, a-3, a-4, and a-5; (**G**) initial transcripts of *LaHDZ34* in WT, a-3, a-4, and a-5; and (**H**) non-cleaved transcripts of *LaHDZ34* in WT, a-3, a-4, and a-5.

The |Ct| value of *LaHDZ31* was the highest in a-4, indicating that more *LaHDZ31* was cleaved in a-4 than that in the other lines (Figure 8A,B). Similarly, the |Ct| value of the initial and full-length transcripts of *LaHDZ32–34* was calculated (Figure 8C–H). The |Ct| value of *LaHDZ32* was the highest in a-4, and that of *LaHDZ33–34* was the highest in a-5. Thus, DEG data of a-4 and a-5 were selected for subsequent analyses.

### 3.6. Pearson’s Correlation Analysis of DEGs Related to the Expression of LaHDZ31–34 in Response to LaMIR166a Overexpression

**Figure 9:**
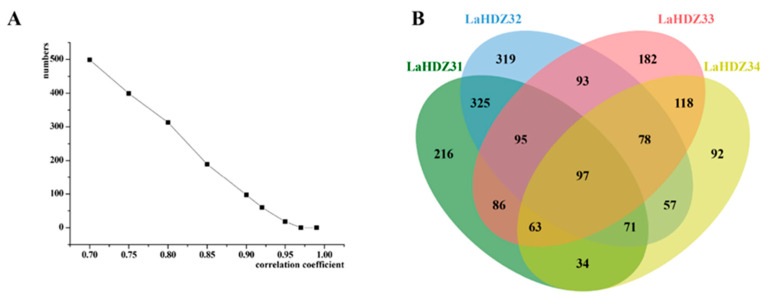
Pearson correlation analysis of related genes, based on the transcriptomic analysis of *Larix kaempferi*. (**A**) Number of genes against correlation coefficients; (**B**) Venn diagram of the 97 genes associated with *LaHDZ31–34* expression; r = 0.9.

TRINITY_DN107458_c0_g1, TRINITY_DN92862_c3_g2, TRINITY_DN100855_c0_g5, and TRINITY_DN95231_c0_g2, which had complete cleavage sites, were identified to belong to *LaHDZ31–34* through sequence alignment analysis. Using the cleaved degree of *LaHDZ31–34*, we predicted that the LaHDZ31–34 TFs play important roles in their corresponding transgenic lines. Therefore, we conducted the Pearson correlation analysis between the expression levels of the corresponding LaHDZ31–34 TFs and DEGs for each cell line. Based on Pearson’s correlation coefficients and the Venn diagram (Figure 9), we selected 97 genes that were significantly associated with all four TFs. 

**Figure 10:**
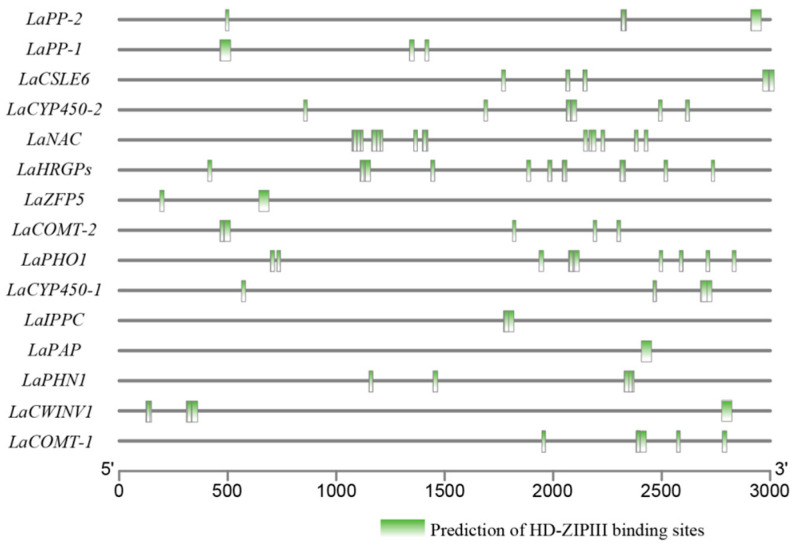
Prediction of HD-ZIP III-binding sites in the promoters of the related genes, based on the transcriptomic analysis of *Larix kaempferi.* Green segments: predicted binding sites.

To further verify the regulatory role of *LaMIR166a*, it is effective to analyze motifs on the promoters of related genes. A region 3000 bp upstream of the 82 genes was used to analyze the *cis*-acting regulatory elements (Table S5). Here, the *LaHDZ31–34* target sites were predicted in 15 genes (Figure 10) using the PlantCARE database, implying that these 15 genes might be regulated by *LaHDZ31–34*. 

TRINITY_DN86651_c0_g1 (*LaZFP5)* was the only downregulated DEG, and the remaining genes were upregulated. In total, 2444 *cis*-acting elements were found in the 15 genes; of these, 316 were involved in the light response (including AE-box, LAMP-element, Box 4, the I-box, the GATA-motif, the G-box, and the GT1-motif), suggesting that the related genes might participate in plant light morphogenesis. Many of the *cis*-acting elements, such as the P-box, TATC-box, TCA-element, ABRE, TGA-element, AuxRR-core, TGACG-motif, and CGTCA-motif, were distributed in the promoter regions. These elements respond to several hormones, including salicylic acid, methyl jasmonate, abscisic acid, gibberellins, and auxin (Table S5).

### 3.7. LaHDZ31–34 Bind to the Promoters of LaPAP, LaPP1, LaZFP5, and LaPHO1

**Figure 11:**
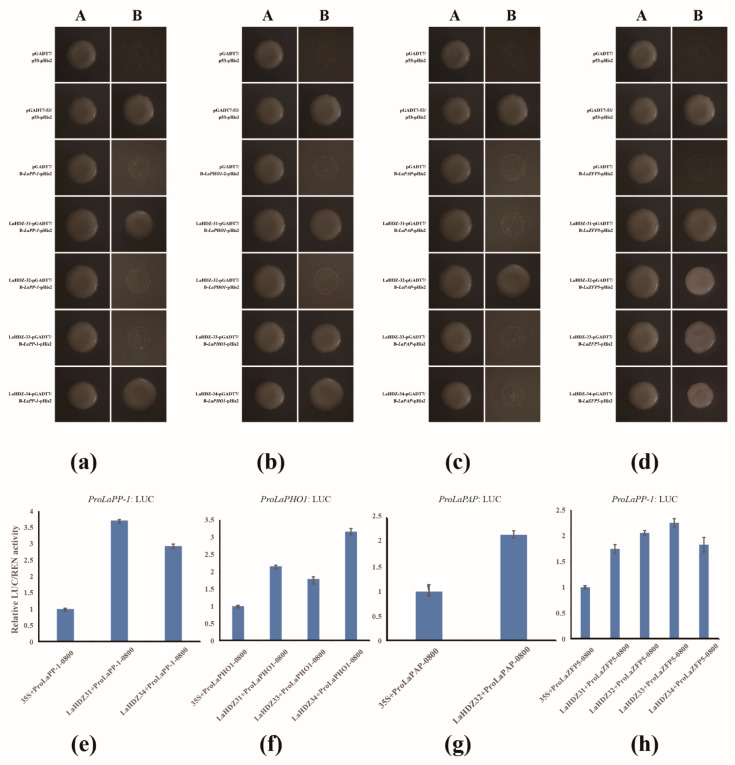
Binding of *LaPP-1*, *LaPHO1*, *LaPAP*, and *LaZFP5* promoters by LaHDZ31–34. Yeast one-hybrid assays of LaHDZ31–34 and the promoters of (**a**) *LaPP-1*, (**b**) *LaPHO1*, (**c**) *LaPAP*, and (**d**) *LaZFP5*, based on the transcriptomic analysis of *Larix kaempferi*. A: SD/-Leu-Trp; B: SD/-Leu-Trp-His containing 30 mmol/L 3-AT. Dual-luciferase assay of LaHDZ31–34 on (**e**) *LaPP-1*, (**f**) *LaPHO1*, (**g**) *LaPAP*, and (**h**) *LaZFP5*.

The Y1H assay was used to examine whether the related genes might be regulated by the LaHDZ31–34 TFs. First, the promoters of the 15 genes were cloned and assigned new names according to gene annotation (Table S6). The 15 genes were then verified by sequencing, and enzyme restriction sites were added to construct vectors; finally, they were transferred into yeast cells. LaHDZ31–34 were able to bind to the promoters of *LaPAP*, *LaPP1*, *LaZFP5*, and *LaPHO1*. In particular, *LaZFP5* was recognized by *LaHDZ31–34* (Figure 11a–d).

Moreover, to further determine the effect of LaHDZ31–34 on the promoter activity of *LaPAP*, *LaPP1*, *LaZFP5*, and *LaPHO1*, dual-LUC assays were performed in tobacco leaves. The results showed that the relative LUC/REN of the experimental group was 1.5 times more than the corresponding control group, which was considered to be positive (Figure 11e–h). These results indicated that LaHDZ31–34 not only bind to the promoter of *LaPAP*, *LaPP1*, *LaZFP5*, and *LaPHO1,* respectively, but also promote their transcription.

## 4. Discussion

The somatic embryogenesis technique applied to Japanese larch is an ideal model for studying the regulation of the early development and morphogenesis of gymnosperms [1]. The complex somatic embryogenesis process includes the following four main stages: the induction of embryogenic tissue, the succession and proliferation of the proembryo group, the induction and maturation of the somatic embryo, and the germination of the somatic embryo. An orderly development process requires the effective regulation of several endogenous signaling molecules. In this study, we focused on the genetic changes generated by *LaMIR166a* overexpression in ESM cell lines. The RNA-Seq transcriptomic analysis was used to reveal differences in the expression of downstream genes. We investigated the roles, regulatory mechanisms and expression of *LaMIR166a* and its target genes *LaHDZ31–34* in *LaMIR166a*-overexpressing transgenic cell lines. This study provides valuable insights into the regulatory network of *miR166*-*HD-ZIP III* in gymnosperms.

The ESM stage provides the necessary physiological signal for embryo formation, and it is pivotal for transformation into an embryo [23]. Morphological differences in ESMs between the *LaMIR166a*-overexpressing lines and WT were associated with differential gene expression, suggesting that critical pathways are active during the cell proliferation process. miRNAs play essential roles during plant development; for example, *miR163* deficiency or overexpression alters secondary metabolite biosynthesis [24]. miRNAs participate in the regulation of secondary metabolite accumulation by regulating their target genes; for example, *miR156* targets *SPL9*, thereby directly regulating anthocyanin biosynthesis [25]. Judicious manipulation of a regulatory gene can increase the activity of an entire biosynthetic pathway [26]. HD-ZIP III, a TF, is involved in stem cell maintenance, meristem growth, and organ morphogenesis in *A. thaliana* [27] and in metabolite biosynthesis. HD-ZIP is associated with terpenoid biosynthesis and is a candidate gene for regulating Eu-rubber accumulation in *Eucommia ulmoides* [28]. Our study provides a considerable amount of cDNA sequence data that could facilitate more detailed studies on the regulatory functions of *LaMIR166a* and help identify the genes related to *LaHDZ31–34*. The sequencing information provides an important resource for studying somatic embryogenesis in *Larix* and other related species. Despite such considerable advantages, the quality of the annotation results would be higher if they could be combined with *L. kaempferi* genomic data. Furthermore, the length and integrity of the sequences obtained using transcriptome splicing affect the annotation percentage. Therefore, further research on this topic is required.

Here, gene expression patterns changed after the overexpression of *LaMIR166a*, 2467 DEGs were detected in the WT and transgenic lines, and the most significant enrichment pathways were associated with secondary metabolites and flavonoid biosynthesis. This suggests that *LaMIR166a* overexpression in ESMs might play an important role in regulating the biosynthesis of secondary metabolites, especially flavonoids. This regulatory relationship should be further verified. We hypothesize that increased flavonoid content in *LaMIR166a*-overexpressing cell lines might affect the development of somatic embryogenesis and thereby affect the germination of somatic embryos.

The results of the cleaved degree of *LaHDZ31–34* revealed that *LaMIR166a*-overexpressing transgenic cell lines might have different gene transfer locations, target sites, and gene copy numbers. Transgenic overexpression cell lines transformed from the same cell line differ, suggesting that the effect of miRNA is crucial. Therefore, it is essential to ensure the consistency of the original system background when using transgenic lines. The expression levels of transformed genes and target genes should be used to select suitable transgenic objects. This has practical importance in Japanese larch production. Interestingly, we found that different cell lines respond differently to *LaMIR166a* overexpression. Our quantitative analysis revealed that *LaMIR166a* played a greater role in the a-4 line, with most of the target genes of *LaHDZ31* and *LaHDZ32* being cleaved; however, *LaHDZ33* and *LaHDZ34* were also cleaved intensively in the a-5 line.

TFs interact with target gene promoters to activate or inhibit target gene function. Correlation analysis can be used to confirm relationships between the regulation of gene expression and related biochemical properties in plants [29,30]. According to GO annotation, 15 genes were divided into two categories, namely, enzymes and transcription factors. Enzymes included hydrolase, oxidoreductase, cellulose synthetase, methyltransferase, phosphatase, and transcription factors including NAC and zinc finger protein. The results of Y1H and dual-LUC assay demonstrated that LaHDZ31–34 bind to the promoters of *LaPAP*, *LaPP1*, *LaPHO1*, and *LaZFP5,* and induce the expression of them. The transcriptome analysis of *LaMIR166a*-overexpressing lines showed that *LaHDZ31–34* and *LaZFP5* transcripts were degraded, and the transcription of *LaPAP*, *LaPP1*, and *LaPHO1* was upregulated, suggesting that *LaHDZ31–34* negatively regulate the expression of *LaPAP*, *LaPP1*, and *LaPHO1.* We speculate that there may be negative feedback regulation. *LaPAP, LaPP1,* and *LaPHO1* are enzyme-related genes and related to the regulation pathway of phosphorus (Pi) deficiency [31], whose expression may be influenced by the pleiotropic effect of *LaMIR166a* overexpression. The zinc finger proteins are involved in gene expression, cell fate specification, and developmental processes, and they can be affected by interactions with nucleic acids. Alternatively, they can directly regulate gene transcription by interacting with proteins [32]. For example, in soybean, SC0F-1 might enhance ABRE-dependent gene expression and cold resistance, mediated by interactions with bZIP TF SGBF-1 [33]. A previous study reported that ZFP5 regulates root hair development in response to inorganic phosphorus (P_i_), and interacts with ethylene signaling [34,35]. In addition, an *Arabidopsis* trichome-related protein could interact with ZFP5 to regulate its downstream genes [36], whereas ZFP5 controls shoot maturation and plays a vital role in regulating inflorescence trichome development in *Arabidopsis* [37]. The Y1H results in the present study demonstrated that LaHDZ31–34 could bind to the promoter of zinc finger protein, suggesting that *LaZFP5* is regulated by *LaHDZ31–34*. The results could facilitate research on the role of ZFP5 in larch root development.

## 5. Conclusions

This study revealed the effects of *LaMIR166a*-overexpressing ESMs on gene regulation. We examined the cleaved degree of *LaHDZ31–34* and the regulatory role of *LaHDZ31–34* with respect to their downstream genes. The differences in gene expression following *LaMIR166a* overexpression might explain the changes in morphogenesis and signaling pathways. *LaMIR166a* overexpression caused *LaHDZ31–34* expression to be downregulated to varying degrees in the transgenic cell lines that we studied. Therefore, the best target should be selected based on the requirements. Selecting the *LaMIR166a*-overexpressing cell lines with the highest responses helped to optimize the transgenic lines. The Y1H and dual-LUC assay revealed that LaHDZ31–34 were able to bind to the promoters of the related genes and that they might play a role in regulating downstream genes. Our findings contribute to a deeper understanding of the regulatory network of *LaMIR166a* and its target genes *LaHDZ31–34*. This provides a basis to improve research on the functions of *LaMIR166a* and theoretical support to achieve better economic benefits from Japanese larch resources.

## Figures and Tables

**Table 1:** Summary of assembly and annotation of the transcriptome.

Sample	Raw Reads (bp)	Clean Reads (bp)	Clean Bases (bp)	GC Percentage (%)	Combined Non-Redundant Unigene (bp)	Total Length (bp)	Mean Length (bp)	N50 (bp)
WT-1	16,885,888	16,764,729	5,065,766,400	46.75	203,256	160,145,128	787.90	996
WT-2	48,658,992	48,169,554	14,597,697,600	46.04				
WT-3	48,260,203	45,731,695	13,719508500	44.83				
WT-4	50,838,449	48,265,826	14,479747800	46.04				
a-3-1	39,548,734	39,163,538	11,864620200	44.85				
a-3-2	23,648,726	23,439,052	7,094,617,800	44.7				
a-3-3	46,286,892	43,875,416	13,162,624,800	45.07				
a-4-1	42,151,448	39,915,863	11,974,758,900	45.69				
a-4-2	36,001,158	34,168,865	10,250,659,500	45.21				
a-4-3	35,848,191	33,974,592	10,192,377,600	45.68				
a-5-1	46,286892	40,263,192	12,078,957,600	46.42				
a-5-2	45,867,514	43,432,516	13,029,800,000	46.19				
a-5-3	52,606,986	49,961,824	14,988,547,200	45.6				

**Table 2:** Summary of the transcriptome annotation.

	Number of Annotated Unigenes	0 ≤ Length < 1000	1000 ≤ Length < 2000	2000 ≤ Length < 3000	3000 ≤ Length < 6000	Length ≥ 6000
GO_Annotation	26,234	13,063	6461	3730	2744	236
KEGG_Annotation	13,546	5245	3767	2417	1916	201
KOG_Annotation	23,331	13,101	5091	2714	2198	227
NR_Annotation	51,321	28,042	11,901	6133	4776	469
NT_Annotation	32,963	14,512	8830	5118	4111	392
Swissprot_Annotation	25,452	11,086	6618	4038	3370	340
All_Annotated	58,487	33,324	13,206	6502	4977	478

**Table 3:** Data of differentially expressed genes (DEGs).

Sample	Number	Downregulated	Upregulated
WT vs. a-3	4354	1786	2568
WT vs. a-4	7441	2724	4717
WT vs. a-5	7314	2958	4356
WT vs. a	5857	2227	3630
DEGs	2467	731	1736

## Data Availability

The data presented in this study are available in Supplementary Materials.

## Supplementary Materials

The following are available online at https://www.mdpi.com/article/10.3390/biology10070576/s1, Table S1: Total unigene numbers assigned to 129 KEGG pathways; Table S2: Statistical analysis of upregulated and downregulated differently expressed genes; Table S3: GO enrichment analysis of DEGs; Table S4: KEGG pathway enrichment in WT, a-3, a-4, and a-5; Table S5: Motif on the promoter of related genes; Table S6: Novel name for Y1H experimental gene; Table S7: Primers used for qRT-PCR; Table S8: Blast search of unigenes against the genome; Table S9: Primers used for PCR and Y1H assay; Table S10: Primers used for dual-luciferase report assays.

## Sample Availability

Samples of the compounds are available from the authors.
